# Internalized HIV stigma, bullying, major depressive disorder, and high-risk suicidality among HIV-positive adolescents in rural Uganda

**DOI:** 10.1017/gmh.2018.15

**Published:** 2018-06-18

**Authors:** S. Ashaba, C. Cooper-Vince, S. Maling, G. Z. Rukundo, D. Akena, A. C. Tsai

**Affiliations:** 1 Mbarara University Science and Technology, Mbarara, Uganda; 2 Massachusetts General Hospital, Boston, MA, USA; 3 Harvard Medical School, Boston, MA, USA; 4 Makerere University College of Health Sciences, Kampala, Uganda

**Keywords:** Adolescents, bullying, depression, HIV, rural Uganda, stigma, suicidality

## Abstract

**Background.:**

Studies conducted in sub-Saharan Africa suggest a high prevalence of depression and suicidality among adolescents living with HIV (ALWH). This is an important public health issue because depression is known to compromise HIV treatment adherence. However, the drivers of depression and suicidality in this population are unclear. We conducted a cross-sectional study to estimate the associations between internalized stigma, bullying, major depressive disorder, and suicidality.

**Methods.:**

We conducted a cross-sectional survey between November 2016 and March 2017, enrolling a consecutive sample of 224 ALWH aged 13–17 years. We collected information on demographic characteristics, internalized HIV-related stigma (using the six-item Internalized AIDS-Related Stigma Scale), bullying victimization (using the nine-item Social and Health Assessment Peer Victimization Scale), major depressive disorder [using the Mini International Neuropsychiatric Interview for Children and Adolescents (MINI-KID)], and suicidality (also using the MINI-KID). We fitted multivariable logistic regression models to estimate the associations between stigma, bullying, major depressive disorder, and suicidality.

**Results.:**

Thirty-seven participants (16%) had major depressive disorder, 30 (13%) had suicidality, and nine (4%) had high-risk suicidality. Ninety-one participants (41%) had high levels of internalized stigma, while 97 (43%) reported two or more bullying events in the past year. In multivariable logistic regression models, major depressive disorder had a statistically significant association with bullying (AOR = 1.09; 95% CI 1.00–1.20; *p* = 0.04); while suicidality (low, moderate, high risk) had statistically significant associations with both bullying (AOR = 1.09; 95% CI 1.01–1.17; *p* = 0.02) and stigma (AOR = 1.30; 95% CI 1.03–1.30; *p* = 0.02).

**Conclusions.:**

Among ALWH in rural Uganda, stigma and bullying are strongly associated with major depressive disorder and suicidality. There is a need to incorporate psychological interventions in the mainstream HIV care to address these challenges for optimal management of HIV among ALWH.

## Introduction

In 2015, approximately 1.8 million adolescents between the ages of 10 and 19 years were living with HIV worldwide (UNAIDS, 2014; Bekker *et al.* 2015), and the number of adolescents living with HIV (ALWH) continues to rise due to increasing availability of HIV antiretroviral medications, which have enabled the survival of perinatally infected children (Brady *et al.* 2010). Numerous studies have documented a high prevalence of depressive symptoms and suicidality among adults living with HIV throughout sub-Saharan Africa (SSA) (Antelman *et al.* 2007; Simbayi *et al.* 2007; Tsai *et al.* 2012; Zunner *et al.* 2015; Ashaba *et al.* 2017; Kinyanda *et al.* 2017). There have been fewer studies of depression and suicidality among ALWH, and these have focused primarily on ALWH in high-income countries (Lyon *et al.* 2000; Mellins *et al.* 2009; Musisi & Kinyanda, 2009; Mellins & Malee, 2013; Betancourt *et al.* 2014; Kim *et al.* 2014). Depression and suicidality are important public health issues not only because of their contribution to the global burden of disease and disability (Murray *et al.* 2012) – with suicide being one of the leading causes of mortality among adolescents worldwide (Gore *et al.* 2011; WHO, 2014) – but also because they are associated with poor HIV treatment adherence and worsened HIV-related outcomes (Antelman *et al.* 2007; Sikkema *et al.* 2010; Tsai *et al.* 2010; Pence *et al.* 2015).

Less is known about the drivers of depression and suicidality among ALWH. HIV is highly stigmatized in SSA (Tsai *et al.* 2013*b*; Tsai, 2015; Chan & Tsai, 2016), and the stigma of HIV has persisted despite the increasing availability of lifesaving antiretroviral medications (Chan *et al.* 2015*a*, *b*). Various forms of HIV stigma have been associated with worsened mental health among HIV-positive persons of all ages (Kinyanda *et al.* 2017) including adolescents (Cluver *et al.* 2008) with consequent adverse impacts on their treatment, health outcomes, and quality of life (Abubakar *et al.* 2016; Dow *et al.* 2016; Fawzi *et al.* 2016; McHenry *et al.* 2016; Vreeman *et al.* 2017). HIV stigma has also been linked to bullying (which can be viewed as a behavioral enactment of stigma) and poor performance in school among ALWH (O'Hare *et al.* 2005; Cluver *et al.* 2010; Boyes & Cluver, 2015). Bullying is defined as intentional, repeated acts of aggressive behavior intended to cause physical or psychological harm and is characterized by an imbalance in power between the perpetrator and the victim (Kochenderfer & Ladd, 1996; Rigby, 2002). Bullying is a particularly noteworthy concern for HIV-positive children across all cultures because adolescence is a critical developmental period during which children value relationships and spend much of their time with peers (Smetana *et al.* 2006; Salmivalli, 2010; Ghoul *et al.* 2013). General population studies of adolescents have linked bullying to multiple mental health problems including depression, anxiety, substance use, and suicidality (Hay *et al.* 2004; Brown *et al.* 2008; Boyes *et al.* 2014).

Despite the public health importance of depression and suicidality among ALWH, there have been relatively few studies examining how these mental health problems are linked to HIV stigma and stigma-related bullying. To date, the bulk of our knowledge about HIV stigma, bullying, and mental health problems among ALWH is derived from a series of large-scale studies of HIV-affected and HIV-positive youth in South Africa by Cluver & Orkin (2009) and Pantelic *et al.* (2017). A major limitation of these studies is their reliance on screening measures of mental health, which in general may yield overestimates of the prevalence of mental disorders, particularly among persons with HIV (Tsai, 2014). To contribute to the existing literature, we conducted a clinic-based, cross-sectional study of ALWH in rural Uganda to estimate the associations between internalized stigma, bullying, major depressive disorder, and suicidality.

## Methods

### Study setting and participants

We conducted the study in Mbarara, a rural region of southwestern Uganda with a population of 195 013 people (UBOS, 2014). We enrolled a consecutive sample of 224 ALWH aged 13–17 years from the HIV clinic attached to the Mbarara Regional Referral Hospital and the Mbarara University of Science and Technology. We excluded adolescents who were not fully aware of their HIV status despite being in HIV care and those who were not physically strong enough to remain present for the duration of the interview. We also excluded those whose cognitive impairments (assessed clinically in consultation with a certified Ugandan psychiatrist) prevented them from fully understanding the interview questions.

### Study measures

We administered all measures using a questionnaire that was translated into the local language (Runyankore). Sociodemographic characteristics were elicited by self-report and included age, sex, level of schooling, duration on antiretroviral therapy, caregiver/family structure (either living with both parents, one parent, grandparents, or siblings), whether the participant was bereaved by the loss of one or both parents, and whether the participant attended a day or boarding school. We also administered the Mini International Neuropsychiatric Interview for Children and Adolescents (MINI-KID, version 6), the internalized stigma scale, and the bullying victimization scale.

The MINI-KID is a short, structured diagnostic interview that is used to obtain valid diagnoses of mental disorders in children and adolescents that are consistent with the Diagnostic and Statistical Manual of Mental Disorders, Fourth edition (DSM-IV) and the International Statistical Classification of Diseases and Related Health Problems (ICD-10) (Sheehan *et al.* 1998, 2010). The MINI-KID module on depression consists of two screening questions, seven additional questions related to depression symptoms, and one question related to functional impairment. The MINI-KID has previously been adapted to obtain valid diagnoses of mental disorders in the Ugandan context (Okello *et al.* 2007; Kinyanda *et al.* 2013; Idro *et al.* 2016; Nalugya-Sserunjogi *et al.* 2016) and was translated into Runyanakore for the purposes of our study. A diploma-level psychiatric clinical officer trained in the diagnosis and management of psychiatric disorders administered the MINI-KID modules on major depressive disorder and suicidality. The suicidality module elicits information about suicidal ideation, planning, and attempts over the past month. The recommended algorithm was applied to the MINI-KID suicidality scores to categorize the study participants as being at low, moderate, or high risk for suicide. A score of 17 or more indicates high-risk suicidality.

HIV stigma was measured using the Internalized AIDS-Related Stigma Scale, a six-item scale designed to measure internalized stigma. It was developed for use among a sample of people living with HIV from the USA, South Africa, and Swaziland (Kalichman *et al.* 2009). It is one of the most widely used stigma measurement scales in the field (Pantelic *et al.* 2015) and has been validated for use in the Ugandan context (Tsai *et al.* 2013*c*). The IARSS items focus on self-blame and concealment of HIV status. Sample items include: ‘it is difficult to tell people about my HIV infection’ and ‘I am ashamed that I am HIV positive’. Each item is scored on a binary response scale (agree/disagree), and the total scale score is computed as the sum of the items, with higher scores representing greater internalized stigma. In the present study, the scale's internal consistency was acceptable, with a Cronbach's *α* of 0.75. A ‘high’ level of internalized stigma was defined as having a total score greater than or equal to the 75th percentile (⩾4).

Bullying victimization was measured using the nine-item Social and Health Assessment Peer Victimization Scale, which elicits experiences of bullying in the past year (Ruchkin *et al.* 2004). The scale was adapted from the Multidimensional Peer Victimization Scale, which showed excellent reliability (*α*  =  0.82) in the initial development study conducted among adolescents in the UK (Mynard & Joseph, 2000) as well as in the studies conducted among black South African children and adolescents (*α* = 0.81) (Cluver *et al.* 2010; Boyes & Cluver, 2015). In the present study, the scale also demonstrated good reliability (*α* = 0.81). The scale contains two items measuring physical bullying victimization, two items measuring verbal bullying victimization, two items measuring relational bullying victimization, two items measuring experience of property damage and theft, and a single item assessing invasion of physical space. The items are scored on a four-point Likert-type scale (never, once, 2–3 times, ⩾4 times). Bullying is defined as having two or more bullying events in the past year. The total bullying victimization score was calculated by summing across all items. At the conclusion of each interview, participants received 10000 Ugandan shillings (approximately US$3 at the time of data collection) to cover transportation costs.

### Ethics review

We interviewed adolescents after obtaining their assent and obtaining written informed consent from a parent/caregiver. Emancipated minors who were below 18 years of age but living independently, and empowered adolescents (i.e. those who were responsible for their HIV care per report of their HIV care provider), provided written informed consent without involvement of their parents/guardians. Research assistants read the consent forms to the participants, who were then given a chance to ask questions for clarification prior to giving their consent. We received ethical approval for our study from the Research Ethics Committee of the Mbarara University of Science and Technology and the Massachusetts General Hospital/Partners Human Research Committee (2016P000482/MGH). Consistent with national guidelines, we also received clearance for the study from the Uganda National Council for Science and Technology (SS4023) and from the Research Secretariat in the Office of the President. Adolescents found to be in acute psychological distress or at acutely elevated risk for suicide, as determined by the psychiatric clinical officer, were referred to the hospital psychiatric ward to receive appropriate care.

### Hypothesis

Based on the literature presented above, we hypothesized that both stigma and bullying would be correlated with the mental health outcomes of interest (major depressive disorder and high-risk suicidality).

### Data analysis

Sample size considerations were driven by the outcome of major depressive disorder. We assumed that the prevalence of major depressive disorder in the sample would be 14%, based on a meta-analysis of major depressive disorder and elevated depression symptom severity among people with HIV in SSA (Tsai, 2014). We further assumed a moderate degree of correlation between the exposures of interest, bullying and stigma, and the other covariates (*R*^2^ = 0.4). With these assumptions, a sample size of 224 would have 80% power to detect an association of 2.0 on the odds ratio scale (Hsieh *et al.* 1998).

We used bivariate analyses to estimate the associations between major depressive disorder and suicidality and the following covariates: sociodemographic characteristics (age, sex, being an orphan, boarding school or day school, serostatus of the caregiver, duration on antiretroviral therapy), bullying, and internalized HIV stigma. We used multivariable logistic regression to estimate their independent correlations with the outcomes, alternately specifying major depressive disorder and suicidality as the dependent variables of interest. We specified high-risk suicidality as the dependent variable in a sensitivity analysis given its clinical importance. To understand the magnitudes of the estimated associations, we calculated the predicted probabilities of major depressive disorder and suicidality evaluated at the 25th and 75th percentiles of the bullying and stigma scores using the delta method. All analyses were conducted in Stata version 13 (StataCorp LP, College Station, Texas, USA).

## Results

We interviewed 224 participants with a mean age of 14.8 years (s.d. 1.4), 131 of whom were female (59%). The mean duration on antiretroviral therapy was 8.5 years (s.d. 4.3). Thirty-seven adolescents (16%) were classified as having major depressive disorder on the MINI-KID. Thirty-one adolescents (14%) were classified as having any (low, moderate, or high risk) suicidality, while nine (4%) were classified as having high-risk suicidality. The mean internalized stigma score was 2.58 (s.d. 1.72). Nearly all participants [201 (90%)] reported at least some degree of internalized stigma, while 91 participants (41%) had ‘high’ levels of stigma as indicated by total scores ⩾4. The mean bullying victimization score was 12.5 (4.99), and 97 participants (43%) reported two or more bullying events in the past year (Table 1).

**Table 1.** Sociodemographic characteristics of the participants (N = 224)

Characteristics	Mean (s.d.) or *N* (%)
Age	14.8 (1.4)
Duration on ART (years)	8.5 (s.d. 4.3)
Distance from the clinic (km)	29.8 (30%)
Sex	
Female	131 (58%)
Male	93 (42%)
Education	
Some primary	38 (17%)
Completed primary	117 (52%)
Some secondary	57 (26%)
Upper secondary	7 (2%)
Tertiary	5 (2%)
Orphan	
Yes	49 (22%)
No	175 (78%)
Type of school	
Day	150 (67%)
Boarding	74 (33%)
Type of caregiver	
Both parents	60 (27%)
Mother alone	79 (35%)
Father alone	22 (10)
Sibling	6 (3%)
Aunt/uncle	22 (10%)
Grand parents	35 (16%)
Serostatus of the caregiver	
Positive	145 (65%)
Negative	28 (12%)
Unknown	51 (23%)
Bullying (two or more bullying events/year)	
Yes	97 (43%)
No	127 (57%)
High level of internalized stigma (score ⩾4)	
Yes	91 (41%)
No	133 (59%)
Suicidality risk	
Low	9 (4%)
Medium	13 (6%)
High	9 (4%)
Major depressive disorder	
Yes	37 (16%)
No	197 (84%)

On bivariate analysis, major depressive disorder was associated with both bullying victimization (OR 1.12; 95% CI 1.04–1.21; *p* = 0.003) and internalized stigma (OR 1.31; 95% CI 1.01–1.73; *p* = 0.04) (Table 2). Any suicidality was also associated with bullying (OR 1.09; 95% CI 1.02–1.16; *p* = 0.008) and internalized stigma (OR 1.33; 95% CI 1.07–1.66; *p* = 0.009).

**Table 2.** Bivariate analysis of factors associated with major depressive disorder and suicidality

	Odds ratio (95% confidence interval), *p* values		
Variable	Major depressive disorder	Any suicidality	High-risk suicidality
Age	0.89 (0.63–1.26), 0.52	0.86 (0.65–1.14), 0.32	0.52 (0.27–0.99), 0.05
Female	1.12 (0.42–3.02), 0.81	2.14 (0.91–5.05), 0.08	1.44 (0.35–5.91), 0.61
Orphan	2.56 (0.93–7.01), 0.07	0.90 (0.35–2.35), 0.83	0.44 (0.05–3.66), 0.45
Boarding school	1.65 (0.63–4.39), 0.31	0.45 (0.17–1.16), 0.09	0.23 (0.03–1.94), 0.18
Stigma	1.31 (1.01–1.73), 0.04	1.33 (1.07–1.66), 0.009	1.47 (1.01–2.16), 0.04
Bullying	1.12 (1.04–1.21), 0.003	1.09 (1.02–1.16), 0.008	1.13 (1.02–1.25), 0.01
HIV-positive caregiver	0.14 (0.02–1.12), 0.06	0.99 (0.42–2.36), 0.98	0.77 (0.15–3.83), 0.75
Duration on ART	1.18 (1.04–1.34), 0.009	0.96 (0.88–1.05), 0.46	0.95 (0.81–1.11), 0.56

On multivariable logistic regression, major depressive disorder had a statistically significant association with bullying (AOR 1.09; 95% 1.00–1.20; *p* = 0.04] but not internalized stigma (AOR 1.27; 95% CI 0.94–1.73; *p* = 0.12) (Table 3). In the regression model predicting any suicidality, both bullying (AOR 1.09; 95% CI 1.01–1.17, *p* = 0.02) and stigma (AOR 1.30; 95% CI 1.03–1.30; *p* = 0.02) had statistically significant associations with the outcome. In a sensitivity analysis, we specified high-risk suicidality as the dependent variable; in this regression model, high-risk suicidality had a statistically significant association with bullying (AOR 1.14; 95% CI 1.01–1.29; *p* = 0.033) but not with stigma (AOR 1.35; 95% CI 0.91–2.01; *p* = 0.13).

**Table 3.** Multivariable regression of the factors associated with depression and suicidality

	Adjusted odds ratio (95% confidence interval), *p* values		
Variables	Major depressive disorder	Any suicidality	High-risk suicidality
Age	0.80 (0.53–1.19), 0.27	0.95 (0.70–1.30), 0.79	0.53 (0.25–1.12), 0.09
Female	0.92 (0.30–2.79), 0.89	2.74 (1.10–6.83), 0.03	2.20 (0.47–10.32), 0.31
Orphan	1.49 (0.47–4.71), 0.49	0.64 (0.27–1.89), 0.42	0.28 (0.03–2.74), 0.27
Boarding school	2.26 (0.71–7.28), 0.16	0.45 (0.16–1.24), 0.13	0.41 (0.04–3.72), 0.42
Bullying	1.09 (1.00–1.20), 0.04	1.09 (1.01–1.17), 0.02	1.14 (1.01–1.29), 0.03
Stigma	1.27 (0.94–1.73), 0.12	1.30 (1.03–1.64), 0.02	1.35 (0.91–2.01), 0.13
HIV-positive caregiver	0.21 (0.02–1.78), 0.15	1.10 (0.42–2.92), 0.84	0.81 (0.13–4.81), 0.81
Duration on ART	1.18 (1.02–1.38), 0.02	0.97 (0.88–1.06), 0.54	0.97 (0.81–1.16), 0.75

The estimated associations were large in magnitude, as suggested by the predictive margins. At the mean of the covariates, the predicted probability of major depressive disorder was 5.2% when evaluated at the 25th percentile of the bullying score and 8.8% when evaluated at the 75th percentile of the bullying score. This difference corresponded to (8.8–5.2)/8 = 45% of the baseline prevalence of major depressive disorder. Similarly, the predicted probability of suicidality was 9.5% when evaluated at the 25th percentile of the bullying score and 14.9% when evaluated at the 75th percentile, or (14.9–9.5)/13 = 41.5% of the baseline prevalence of suicidality. The estimated associations were slightly larger for internalized stigma. The predicted probability of major depressive disorder was 5.8% when evaluated at the 25th percentile of the stigma score and 10.0% when evaluated at the 75th percentile of the stigma score, corresponding to (10–5.8)/8 = 52.5% of the baseline prevalence. The predicted probability of suicidality was 8.6% when evaluated at the 25th percentile of the stigma score and 16.9% when evaluated at the 75th percentile, or (16.9–8.6)/13 = 63.4% of the baseline prevalence of suicidality.

## Discussion

In this cross-sectional, clinic-based study of HIV-positive adolescents, we found that HIV stigma and bullying were associated with major depressive disorder and high-risk suicidality. The estimated associations were statistically significant, large in magnitude, and robust to potential confounding by several measured covariates. While several previously published studies based on the data collected in SSA have examined the correlations between these constructs (Hay *et al.* 2004; Brown *et al.* 2008; Cluver & Orkin, 2009; Boyes *et al.* 2014; Pantelic *et al.* 2017), our study adds to this literature by using structured diagnostic interviews to assess for major depressive disorder and high-risk suicidality. Our findings have important policy and programmatic implications for the holistic care of HIV-positive children and adolescents and suggest a need for integrating mental health care into mainstream HIV clinic care and for the development of interventions that aim to avert stigma and bullying associated with HIV (Vreeman *et al.* 2017).

We found that bullying victimization was associated with both major depressive disorder and high-risk suicidality. Internalized stigma was also associated with both major depressive disorder and suicidality, although the estimated associations remained statistically significant after multivariable adjustment only in the analysis predicting any suicidality (but not major depressive disorder or high-risk suicidality). These findings are consistent with the findings from previous studies showing increased risks of poor mental health in the setting of bullying among HIV-positive and HIV-affected children and adolescents (Cluver *et al.* 2010; Boyes & Cluver, 2015; Pantelic *et al.* 2017). Although bullying has also been reported to be a predictor of internalizing symptoms among children and adolescents in the general population (Hay *et al.* 2004; Brown *et al.* 2008; Cluver & Orkin, 2009), there is a need for elevated concern about bullying of HIV-positive/HIV-affected youth given that the stigma of HIV is frequently a risk factor for bullying itself (O'Hare *et al.* 2005; Cluver *et al.* 2010; Boyes & Cluver, 2015). In the setting of HIV, bullying can be considered a behavioral manifestation of stigma also described as enacted stigma, which is consistent with previously published work linking HIV stigma and mental health problems among children and adolescents (Cluver *et al.* 2008; Norcini Pala *et al.* 2015).

In terms of other ancillary findings, we found that suicidality was more prevalent among girls *v*. boys. This finding mirrors previously published findings showing that suicidality is more prevalent among HIV-positive women in SSA (compared with HIV-positive men) (Schlebusch & Vawda, 2010; Chikezie *et al.* 2012; Kinyanda *et al.* 2012), as well as the well-known finding that depression is more prevalent among women *v*. men generally (Weissman & Klerman, 1977). In Uganda as well as other countries in SSA, HIV incidence and prevalence are higher among women (Uganda AIDS Indicator Survey (UAIS), 2014; Patra & Singh, 2015; Sia *et al.* 2016) and HIV-positive women are also more vulnerable to HIV-related stressors that predispose them to internalizing the stigma of HIV compared with men (Rohleder & Gibson, 2006). A second notable finding is that lack of social support was associated with major depressive disorder. This finding is consistent with previously published findings showing that social support is protective against mental health problems among HIV-affected adolescents and youth (Lee *et al.* 2007; Cheng *et al.* 2014; Vreeman *et al.* 2017) and among HIV-positive adults (McDowell & Serovich, 2007; Tsai *et al.* 2012; Casale *et al.* 2015). Third, we found that increasing duration on HIV treatment was positively associated with the odds of major depressive disorder. This finding was unexpected, as it stands in contrast to several previously published studies showing that HIV treatment is associated with a reduction in depression symptom severity among HIV-positive adults (Wagner *et al.* 2012; Tsai *et al.* 2013*a*; Martinez *et al.* 2014*b*). It should be noted, however, that the duration of treatment was determined by self-report rather than by review of clinic records, which may limit its accuracy.

Of note is the fact that our sample consisted of adolescents who were on antiretroviral therapy for an average of more than 8 years. Although ALWH engaged in care have access to support, and HIV treatment in general has been shown to diminish stigma (Seeley & Russell, 2010; Campbell *et al.* 2011; Mbonye *et al.* 2013; Tsai *et al.* 2013*a*), our findings indicate that ALWH still experience persistent internalized HIV stigma, bullying, and other mental health problems including depression even when on long-term antiretroviral therapy (Martinez *et al.* 2014*a*; Treves-Kagan *et al.* 2016). As such there is a room to enhance psychological services for adolescents in HIV care to enhance antiretroviral treatment adherence and improve treatment outcomes (Dow *et al.* 2016; McHenry *et al.* 2016; Vreeman *et al.* 2017). Recent work from Uganda has shown that evidence-based psychological interventions can be tailored to the local context to reduce stress and depression among persons with HIV (Nakimuli-Mpungu *et al.* 2014*a*, *b*).

Interpretation of our study findings should take into account a number of limitations. The cross-sectional study design does not allow for the evaluation of the causal relationship between stigma, bullying, depression, and suicidality. Second, our sample consisted solely of ALWH aged 13–17 years, so our results may not be generalizable to younger children and or older youth. Third, this study was based on a consecutive sample of ALWH who were receiving care in a well-structured HIV clinic. Fourth, the majority of study participants had been on treatment for an extended duration, suggesting they were perinatally infected. Therefore, these findings may not generalize to HIV-positive adolescents who have acquired HIV behaviorally. There is some evidence to suggest that the stigma attached to behaviorally acquired HIV is more severe (Orban *et al.* 2010) and may therefore have more severe impacts on mental health.

## Conclusion

Our findings show that stigma and bullying are common among HIV-positive adolescents engaged in HIV care in rural Uganda and that these are strong predictors of depression and suicidality. These findings have important bearing on public health impact since stigma, bullying victimization, and depression affect the ability of affected individuals to seek care and adhere to treatment, ultimately resulting in poor physical outcomes. Bearing in mind that our sample consisted of adolescents who had been in HIV care for an average of 8 years yet nonetheless continued to experience psychological distress and other challenges of stigma and bullying indicates that availability of antiretroviral medications and support from HIV care providers is not enough (Chan *et al.* 2015*a*, *b*; Chan & Tsai, 2016; Treves-Kagan *et al.* 2016). There is a need to incorporate psychological interventions into mainstream HIV care to address these challenges for optimal HIV treatment among ALWH. Additionally, interventions to promote awareness in schools may also be effective in reducing stigma and promoting support for ALWH among peers.
